# Relationship between cortical activation and sleep quality in cerebral small vessel disease patients: a functional near-infrared spectroscopy (fNIRS) study

**DOI:** 10.3389/fneur.2025.1618240

**Published:** 2025-09-01

**Authors:** Yuwei Mi, Yu Wang, Jieqiong Hu, Shanshan Hua, Lanlan Wang, Jianhong Yang, Xiaoqin Zhang, Yunxin Ji

**Affiliations:** ^1^Department of Psychosomatic Medicine, The First Affiliated Hospital of Ningbo University, Ningbo, China; ^2^Department of Radiology, The First Affiliated Hospital of Ningbo University, Ningbo, China; ^3^Department of Neurology, The First Affiliated Hospital of Ningbo University, Ningbo, China; ^4^Ningbo University Medical School, Ningbo, China

**Keywords:** cerebral small vessel disease, sleep disturbance, fNIRS, verbal fluency task, prefrontal cortex

## Abstract

**Background:**

Sleep disturbance is common in patients with cerebral small vessel disease (CSVD). The impact of insomnia on cortical activation in CSVD patients and its association with sleep quality remains unclear. Our study used functional near-infrared pectroscopy (fNIRS) to investigate differences in cortex activation in CSVD patients with sleep disturbance (CSVD + S) and CSVD patients without sleep disturbance (CSVD − S) during the verbal fluency task (VFT), and further explored its relationship with sleep quality.

**Methods:**

59 CSVD + S and 69 CSVD − S matched for age, gender, and educational level were recruited. Sleep quality was assessed by the Pittsburgh Sleep Quality Index (PSQI). fNIRS was used to assess frontotemporal activation in CSVD patients during the VFT.

**Results:**

The prevalence of sleep disturbance in CSVD patients was 46.01%. Compared to CSVD − S, CSVD + S exhibited lower cortex activation in the bilateral medial prefrontal cortex (mPFC) and dorsolateral prefrontal cortex (DLPFC) (false discovery rate corrected *p* < 0.05). Correlation analysis showed bilateral mPFC and DLPFC activation negatively correlateed with PSQI scores in CSVD patients. Further stepwise multiple linear regression found right mPFC activation had the strongest negative correlation with PSQI scores after adjusting for confounding factors.

**Conclusion:**

Our study used fNIRS to demonstrate that CSVD patients with sleep disturbance showed poorer prefrontal cortex activation during the VFT, which is associated with poorer sleep quality.

## 1 Introduction

Cerebral small vessel disease (CSVD) is a syndrome caused by various etiologies affecting intracranial small vessels (arterioles, capillaries, and venules), leading to clinical, imaging, and pathological manifestations (1). The typical imaging markers of CSVD include lacunes of presumed vascular origin, cerebral microbleeds (CMBs), enlarged perivascular spaces (EPVS), white matter hyperintensities (WMHs) of presumed vascular origin, and brain atrophy (2). The incidence rate and severity of CSVD are closely related to age (3). With the aging of the population, the issue of CSVD combined with sleep disturbance has attracted more and more attention (4–6).

Sufficient and good-quality sleep is essential for maintaining human life activities and serves as an indispensable part of preserving normal central nervous system functions. On the contrary, poor sleep quality not only has a significant negative impact on physical health, such as cardiovascular and cerebrovascular diseases (7), but also has negative effects on cognitive function (8) and emotional regulation (9). Sleep disturbance is one of the common health issues among older adults (10), and the pooled prevalence of sleep disturbance among the elderly is 35.9% (11). Recent studies have found that the prevalence of sleep disturbance in elderly CSVD patients is approximately 46–50% (12, 13), which is generally higher than among the elderly. Additionally, poor sleep quality is associated with WMHs severity (4), EPVS in the basal ganglia region (5), and lower cortical brain volume (14, 15), indicating a relationship between CSVD and sleep disturbance. A magnetic resonance imaging (MRI) study revealed that CSVD patients with sleep disturbance exhibited structural brain alterations compared to those without sleep disturbance, and the gray matter volume of the right caudate and bilateral calcarine cortex was correlated with sleep quality in CSVD patients (16). Another DTI study found that compared with those without sleep disturbance, CSVD patients with sleep disturbance had more damage to white matter microstructural damage in sleep related brain regions such as the frontal lobe (13). Current neuroimaging research primarily focuses on structural brain abnormalities in CSVD patients with sleep disturbances, whereas functional neuroimaging research remains limited. Therefore, assessing brain function is crucial for understanding the pathophysiology of sleep disturbance in CSVD patients.

Functional near-infrared spectroscopy (fNIRS), an emerging functional neuroimaging modality, has garnered increasing attention in recent years as a safe and non-invasive method for monitoring cerebral hemodynamics. It utilizes the differences in near-infrared light absorption rates at different wavelengths of 650–1,000 nm between oxyhemoglobin (Oxy-Hb) and deoxygenated hemoglobin (Deoxy-Hb) in brain tissue (17, 18). fNIRS distinguishes itself in neuroimaging through dual comparative advantages, it achieves superior spatial resolution to electroencephalography (EEG) while simultaneously offering enhanced temporal resolution over functional magnetic resonance imaging (fMRI) (18). Furthermore, this modality demonstrates superior motion artifact tolerance compared to conventional neuroimaging approaches, coupled with cost-effectiveness, user-friendly portability, and simplified operational requirements (18). Currently, fNIRS has been widely used to evaluate brain function in patients with neuropsychiatric disorders.

The combination of neuropsychological measurements and brain imaging techniques can provide a perspective on real-time brain functional status during tasks. The verbal fluency task (VFT) is a well-established neuropsychological paradigm in which participants must participate in executive-mediated cognitive operations (including working memory maintenance, inhibitory control allocation, and attentional regulation) to effectively process phonemic information through systematic vocabulary retrieval strategies (19). The combination use of fNIRS and VFT has become a common approach for studying neuropsychiatric disorders such as CSVD, insomnia, and major depressive disorder (MDD). A fNIRS study compared two CSVD patients with one healthy control, and found abnormal hemodynamic responses in CSVD patients during the VFT (20). A fNIRS study found that chronic insomnia patients showed hypoactivation in the bilateral medial prefrontal cortex (mPFC) and dorsolateral prefrontal cortex (DLPFC) during VFT compared to healthy controls (21). Another fNIRS study also found that bilateral mPFC and DLPFC activation was lower in MDD patients with insomnia than in those without insomnia (22). To the best of our knowledge, no published studies have employed the fNIRS to evaluate cortical function differences between CSVD patients with and without sleep disturbance. In addition, the relationship between cortical function and sleep quality in CSVD patients is still unclear.

To address this research gap, our study used the fNIRS to investigate the differences in cortical activation of the frontotemporal regions between CSVD patients with and without sleep disturbance during the VFT, and further explored the relationship between cortical activation and sleep quality in CSVD patients. We speculate that (1) CSVD patients with sleep disturbance would have lower cortical activation levels than those without sleep disturbance; (2) there would be a correlation between cortical activation and sleep quality in CSVD patients.

## 2 Materials and methods

### 2.1 Participants

This cross-sectional study was conducted between November 2023 and December 2024, at neurology outpatient department of the First Affiliated Hospital of Ningbo University. The study protocol was registered at Chictr.org.cn under the number ChiCTR2500098161. The Ethics Committee of the First Affiliated Hospital of Ningbo University (no. 2023-103A) approved this study. All participants signed an informed consent form before entering this study.

The inclusion criteria for CSVD patients were as follows: (1) aged 18–75 years; (2) right-handed Han Chinese; (3) total CSVD burden ≥ 1. The total CSVD burden was quantified as an ordinal scale (0–4 points) (Supplementary Table 1), where one point was allocated per marker: ① presence of lacunes ≥ 1; ② CMBs, ≥1; ③ moderate-to-severe EPVS (grade 2–4) in the basal ganglia; ④ deep WMHs graded as Fazekas score 2 (beginning confluence) or 3 (confluent lesions), or irregular periventricular WMHs with deep white matter extension (Fazekas score 3) (23).

Exclusion criteria were as follows: (1) intracranial or extracranial arteries stenosis ≥ 50%, or prior carotid endarterectomy; (2) genetic related CSVD, such as cerebral autosomal dominant angiopathy with subcortical infarcts and leukoencephalopathy (CADASIL); (3) WMHs of nonvascular etiology, such as multiple sclerosis and neoplasms; (4) presence of atrial fibrillation or other serious physical illnesses; (5) alcohol or drug abuse disorders or severe mental disorders; (6) no other sleep related medical history, such as sleep breathing disorders (such as obstructive sleep apnea) and sleep related movement disorders (such as periodic limb movement disorders); (7) use of antidepressants or sedative-hypnotics medications in the past month; (8) claustrophobia or inability to tolerate magnetic resonance imaging (MRI) procedures due to severe anxiety; (9) difficulties with communication.

The sample size was calculated using the following standard formula for our study: *n* = Z^2^ (1-*p*)/*ε*^2^*p*. n, required sample size; *p*, expected prevalence; Z, 95% confidence interval, equal to 1.96; ε, marginal error, equal to 0.1 (10%). A previous study has found that approximately 46% of CSVD patients experience insomnia, thus the expected prevalence *p* was set at 0.46 (13). Substituting these values into the formula yielded a minimum required sample size of 112 participants. Considering a 10% non-response rate, the final target sample size was set at 125 participants to ensure statistical adequacy.

**Figure 1:**
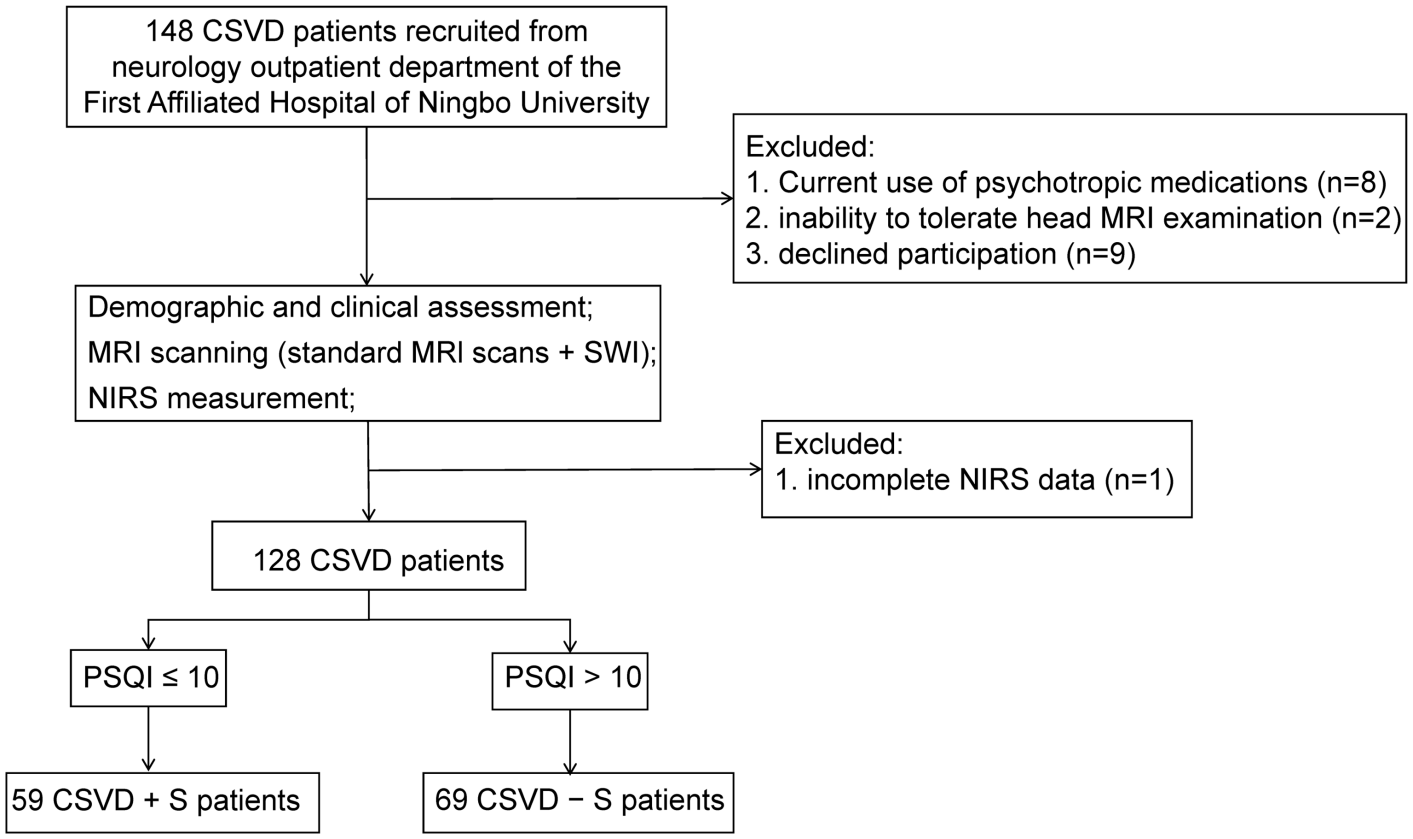
A flowchart of recruitment and exclusion of patients with CSVD. CSVD, cerebral small vessel disease; NIRS, near-infrared spectroscopy; MRI, magnetic resonance imaging; SWI, susceptibility weighted imaging; PSQI, Pittsburgh Sleep Quality Index; CSVD + S, CSVD with sleep disturbance; CSVD − S, CSVD without sleep disturbance.

A total of 148 CSVD outpatient patients were screened, of which 20 patients were excluded due to the following reasons: incomplete near-infrared spectroscopy (NIRS) data (*n* = 1), current use of psychotropic medications (antidepressants/sedative-hypnotics, *n* = 8), inability to tolerate head MRI examination (*n* = 2), and declined participation (*n* = 9). Finally, our study recruited a total of 128 CSVD patients (Figure 1).

### 2.2 Demographic data

A self-designed questionnaire was administered by trained research staff to collect general information, including age, gender, body mass index (BMI), and years of education, and vascular risk factors, including hypertension (yes/no), diabetes mellitus (yes/no), hypercholesterolemia (yes/no), smoking status (current/former/never), and alcohol consumption (current/former/never). BMI (kg/m^2^) was calculated as weight (kg) divided by the square of height (m).

### 2.3 Clinical assessments

The Pittsburgh Sleep Quality Index (PSQI) is a self-report questionnaire commonly used to assess sleep quality over the past month. The questionnaire consists of 19 items organized into seven components: subjective sleep quality, sleep latency, sleep duration, sleep efficiency, sleep disturbance, use of hypnotic drugs, and daytime dysfunction. Each component scores from 0 to 3 points, and the total score is the sum of the seven components, ranging from 0 to 21. A higher score indicates poorer sleep quality (24). The PSQI score is correlated with the Insomnia Severity Index (25). In our study, patients with PSQI scores >10 were classified as CSVD with sleep disturbance (CSVD + S), while those with scores ≤10 were classified as CSVD without sleep disturbance (CSVD − S) (22).

The Chinese version of the Mini-Mental State Examination (MMSE) was used to assess global cognitive function of all CSVD patients. The total score is 30 points, and the cognitive domains test includes orientation, memory, attention and calculation, execution, language, and visual spatial abilities (26). The MMSE score is highly correlated with education, and a higher score indicates better cognitive function.

All CSVD patients completed standard cranial MRl scans (including T1-weighted, T2-weighted, T2-fluid-attenuated inversion recovery, and diffusion-weighted imaging sequences) and susceptibility weighted imaging (SWI) examinations. All imaging sequences were independently reviewed and evaluated by two radiologists who had received standardized training and possessed over five years of clinical experience. In cases of discrepancies between the two reviewers’ assessments, a third senior radiologist provided the final determination. The MRI markers of CSVD were defined according to the STRIVE-2 consensus criteria (27). Lacunes were characterized as subcortically located, CSF-isointense cystic lesions (3–15 mm in diameter) exhibiting a hyperintense peripheral rim on MRI. CMBs were identified as well-circumscribed, rounded foci of SWI signal voids measuring 2–10 mm in diameter. EPVS were defined as punctate, round/ovoid, or linear structures (typically <3 mm in diameter) demonstrating isointensity to cerebrospinal fluid across all MRI sequences. WMH displayed abnormal signals of different sizes in brain white matter regions, and the severity of the WMHs was assessed using the Fazekas scale. As previously described, we quantify the total CSVD burden based on these four imaging markers (Supplementary Table 1). We conducted a consistency assessment of the results evaluated by the first two radiologists, and the Kappa consistency coefficient of the CSVD total burden score was 0.937, indicating strong consistency among evaluators.

### 2.4 Verbal fluency task

**Figure 2:**
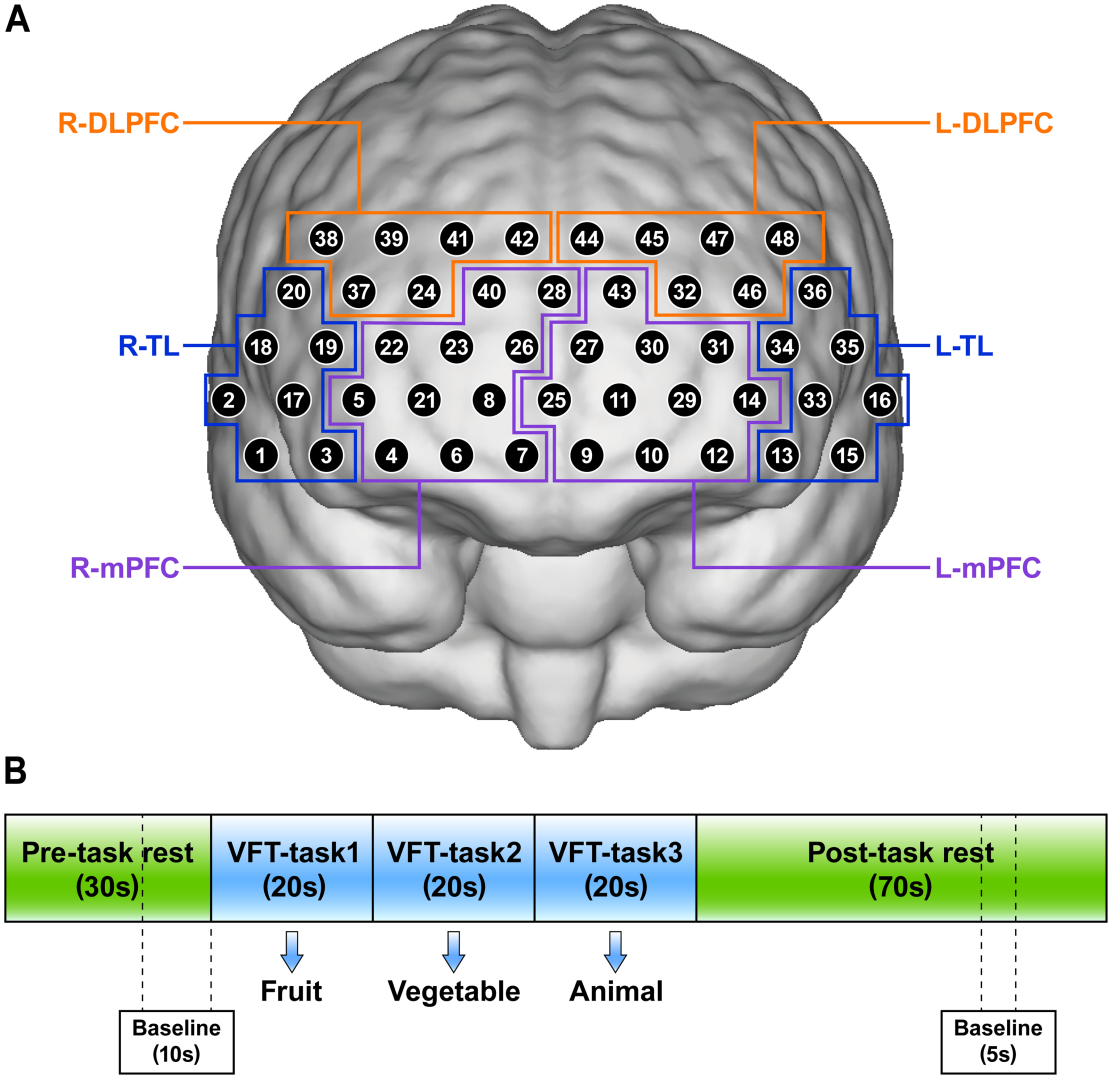
**(A)** The position of 48 channels and definition of regions of interests (ROIs). **(B)** Verbal fluency task (VFT) for near-infrared spectroscopy. R, right; L, left; TL, temporal lobe; mPFC, medial prefrontal cortex; DLPFC, dorsolateral prefrontal cortex.

In our study, we evaluated cognitive function using the VFT, which consists of a 30-s pre-task rest period, a 60-s task period, and a 70-s post-task rest period (Figure 2B). During the pre-task and post-task rest periods, participants were asked to repeat counting from numbers 1 to 5. During the task period, participants were instructed to verbally generate as many items as possible in response to three semantic categories (“fruits,” “vegetables,” and “animals”), with each cue allocated a 20-s response period (28).

fNIRS data acquisition was performed between 8:30–10:30 AM under standardized environmental conditions. Prior to initiating the VFT, window drapes were secured to standardize ambient illumination and mitigate confounding effects from external environmental variables. Participants received explicit instructions to maintain an upright seated posture with minimal voluntary motion. Trained research staff documented the accuracy of responses to the three verbal stimuli, with this quantitative measure constituting the final VFT performance score.

### 2.5 NIRS measurement

The concentration changes of Oxy-Hb in the brains of participants were measured using a multi-channel near-infrared optical imaging system NirScan-6000C (NirScan, Danyang Huichuang Medical Equipment Co., Ltd., China) during the VFT. The device had a total of 48 effective channels, consisting of 15 light sources and 16 detectors, and can cover the bilateral frontal and temporal cortex with a fixed channel spacing of 3.0 cm (referred to as the 10/20 international system). Data acquisition was performed at a sampling rate of 11 Hz, utilizing a triple-wavelength light source (730 nm, 808 nm, and 850 nm).

Referring to previous literature (21, 22), we set 6 regions of interests (ROIs): the right temporal lobe (R-TL), left temporal lobe (L-TL), right mPFC (R-mPFC), left mPFC (L-mPFC), right DLPFC (R-DLPFC), and left DLPFC (L-DLPFC). The position of 48 channels and the corresponding brain regions are shown in Figure 2A.

### 2.6 Data preprocessing and analysis

**Figure 3:**
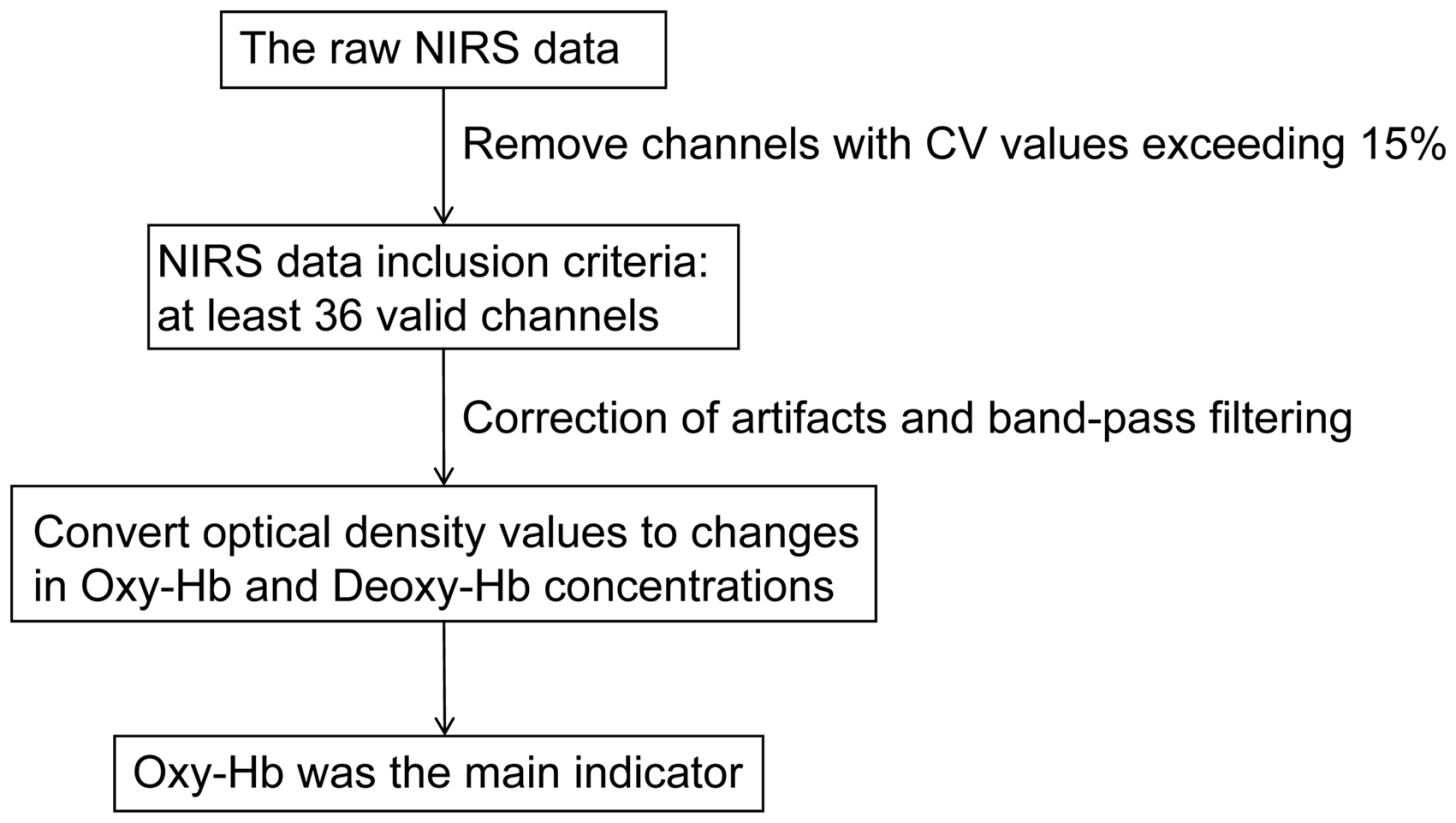
A flowchart of NIRS data preprocessing. NIRS, near-infrared spectroscopy; CV, coefficient of variation; Oxy-Hb, oxyhemoglobin; Deoxy-Hb, deoxygenated hemoglobin.

We utilized the NirSpark software package (NirSpark, Danyang Huichuang Medical Equipment Co., Ltd., China) for the analysis and processing of raw NIRS data. The raw data underwent preprocessing through the following sequential steps (Figure 3). Firstly, we calculated the relative coefficient of variation (CV, %) to evaluate the signal-to-noise ratio (SNR) performance of the channels, and removed channels with CV values exceeding 15% (29). NIRS data inclusion criteria mandated at least 36 valid channels per subject, spanning prefrontal cortex with bilateral temporal lobe coverage (21). Secondly, correct artifacts caused by motion through moving SD combined with cubic spline interpolation algorithms. A band-pass filter with a frequency of 0.01–0.20 Hz was used to remove physiological noise (such as respiration and cardiac activity) to ensure the stability and accuracy of the results. Finally, the modified Beer–Lambert law was used to convert optical density values to changes in the Oxy-Hb and Deoxy-Hb concentrations. In our study, Oxy-Hb was selected as the primary analytical parameter over Deoxy-Hb due to its superior SNR (30, 31).

VFT block waveforms were calculated with a block range set of 0–125 s, with a pre-baseline range of 0–10 s and a post-baseline range of 120–125 s. Linear fitting was used to the two baselines data. We use a 60-s task period for constructing words as a time window to analyze mean Oxy-Hb changes. The Oxy-Hb concentration variations underwent spatial averaging across all optical measurement channels within individual regions of interest (ROIs).

### 2.7 Statistical analysis

First, the normality of continuous variable data was tested using the Shapiro–Wilk test. Normally distributed continuous variables were expressed as mean ± standard deviation (mean ± SD), while non-normally distributed continuous variables were expressed as median (quartiles) [M (Q1, Q3)]. Categorical variables were represented as frequencies and percentage [*n* (%)]. Second, for continuous variables, differences between groups were analyzed as follows: for normally distributed data with homogeneous variance, an unpaired t test was employed; For normally distributed data with heterogeneous variance, an unpaired t test with Welch’s correction was used; For data that does not follow a normal distribution, the Mann–Whitney U test was applied. As for categorical variables, the chi-square test was used to compare differences between the two groups. Third, false discovery rate (FDR) was adopted for correcting multiple comparisons in the data of mean Oxy-Hb changes in channels and ROIs. Forth, spearman correlation analysis was used to examine the association between cortical activation and PSQI scores in CSVD patients. Finally, significant different ROIs activation variables were entered into a stepwise multiple linear regression model to explore which brain regions had the strongest association with sleep quality in CSVD patients. In this analysis, age, gender, BMI, and education year were adjusted in model 1. Further adjustments were made for CSVD total burden, VFT and MMSE in model 2. All statistical analyses were performed using SPSS 26.0 (SPSS Inc., Chicago, IL, United States). Figures and graphs were made with GraphPad Prism 8 and Adobe Illustrator software. All *p*-values were two-tailed with a significance level set at 0.05.

## 3 Results

### 3.1 Demographic and clinical characteristics

Table 1 summarizes the differences in demographic and clinical characteristics between the CSVD + S group (*n* = 59) and CSVD − S group (*n* = 69). The prevalence of sleep disturbance in CSVD patients was 46.01%. No statistically significant differences between the two groups in terms of demographic factors (including age, gender, BMI, and education), vascular risk factors (including hypertension, diabetes mellitus, hypercholesterolemia, smoking status, and alcohol consumption), and imaging characteristics (including total Fazekas score, Lacunes, CMBs, EPVS, and total CSVD burden) (all *p* > 0.05).

**Table 1:** Comparison of demographic and clinical characteristics between CSVD patients with and without sleep disturbance.

Characteristic	CSVD + S (*n* = 59)	CSVD − S (*n* = 69)	t/Z/χ^2^	*p*-value
Demographic factors				
Age (years)	64 (57, 67)	61 (56, 66)	−1.171	0.242
Gender (male, %)	20 (33.90)	31 (44.90)	1.614	0.204
BMI (kg/m^2^)	23.60 ± 3.17	23.78 ± 2.89	0.332	0.740
Education (years)	7 (3, 9)	8 (6, 9)	−1.397	0.163
Vascular risk factors (%)				
Hypertension	36 (61.02)	32 (46.38)	2.737	0.098
Diabetes Mellitus	6 (10.17)	3 (4.35)	1.649	0.199
Hypercholesterolemia	34 (57.63)	46 (66.67)	1.109	0.292
Smoking status			3.574	0.167
Current	5 (8.47)	7 (10.14)		
Former	4 (6.78)	12 (17.39)		
Never	50 (84.75)	50 (72.46)		
Alcohol consumption			4.582	0.101
Current	5 (8.47)	6 (8.70)		
Former	8 (13.56)	20 (28.99)		
Never	46 (77.97)	43 (62.32)		
Neuropsychological tests				
PSQI	13 (12, 16)	7 (5, 9)	−9.757	<0.001
MMSE	27 (24, 29)	29 (26.5, 29)	−2.843	0.004
VFT	16 (13, 19)	19 (16, 22)	−2.664	0.008
Imaging characteristics				
Total Fazekas score	3 (2, 4)	3 (2, 4)	−0.626	0.531
Lacunes ≥1, *n* (%)	15 (25.42)	20 (28.99)	0.203	0.652
CMBs ≥1, *n* (%)	7 (11.86)	15 (21.74)	2.179	0.140
EPVS	13 (8, 19)	15 (8, 23.5)	−0.904	0.366
Total CSVD burden	2 (1, 2)	2 (1, 2)	−0.152	0.880

CSVD, cerebral small vessel disease; CSVD + S, CSVD with sleep disturbance; CSVD − S, CSVD without sleep disturbance; BMI, body mass index; PSQI, Pittsburgh sleep quality index; MMSE, mini-mental state examination score; VFT, verbal fluency task; CMBs, cerebral microbleeds; EPVS, enlarged perivascular spaces.

There were statistically significant differences in the neuropsychological tests between the two groups. The total PSQI score of the CSVD + S group was significantly higher than that of the CSVD − S group (Z = −9.757, *p* < 0.001), while the CSVD + S group demonstrated significantly lower cognitive performance compared to the CSVD − S group, as evidenced by reduced MMSE scores (Z = −2.843, *p* = 0.004) and fewer word responses during the VFT (Z = −2.664, *p* = 0.008).

### 3.2 Mean Oxy-Hb changes of different channels during the VFT

**Figure 4:**
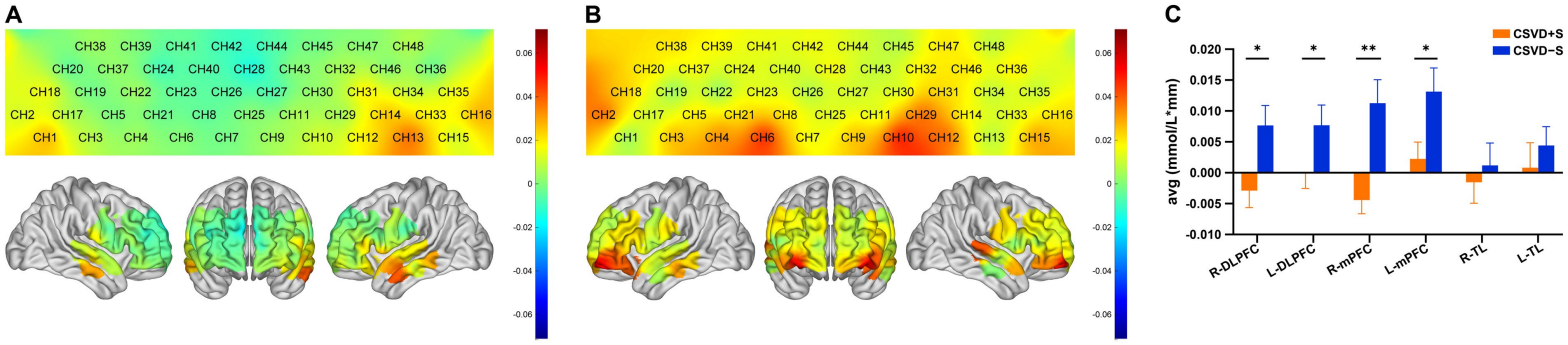
**(A)** Activation map of the CSVD + S group in each channel based on the block average. The color scale depicts the change of Oxy-Hb value range from −0.7 to 0.7 in mmol/L*mm. **(B)** Activation map of the CSVD − S group in each channel based on the block average. The color scale depicts the change of Oxy-Hb value range from −0.7 to 0.7 in mmol/L*mm. **(C)** Cortical activation in different ROIs (*p*-values for FDR correction); data was expressed as mean ± standard error of the mean. CSVD, cerebral small vessel disease; CSVD + S, CSVD with sleep disturbance; CSVD − S, CSVD without sleep disturbance; Oxy-Hb, oxyhemoglobin; ROIs, regions of interests; FDR, false discovery rate; R, right; L, left; TL, temporal lobe; mPFC, medial prefrontal cortex; DLPFC, dorsolateral prefrontal cortex. * 0.01 < *p* ≤ 0.05, ** 0.001 < *p* ≤ 0.01.

Figures 4A,B present the hemodynamics changes of the Oxy-Hb in different channels during the VFT of the CSVD + S group and the CSVD − S group, respectively. During the VFT, the CSVD + S group exhibited significantly lower mean Oxy-Hb concentrations at channels 6, 8, and 28 (mainly located in the R-mPFC), 10 (mainly located in the L-mPFC), 42 (mainly located in the R-DLPFC) compared to the CSVD − S group (FDR corrected *p* < 0.05; Table 2).

**Table 2:** Channels with significant differences of mean Oxy-Hb changes between CSVD with and without sleep disturbance.

Channel	Location	Mean Oxy-Hb changes in 60s task period (mmol/L*mm)		t/Z	Unadjusted, *p*-value	*p*-value (FDR corrected)
		CSVD + S	CSVD − S			
6	R-mPFC	−0.009 (−0.025, 0.010)	0.017 (0, 0.045)	−4.244	<0.001	<0.001
8	R-mPFC	−0.010 (−0.023, 0.015)	0.011 (−0.012, 0.038)	−3.226	0.001	0.002
10	L-mPFC	0.002 (−0.020, 0.032)	0.033 (0.002, 0.059)	−3.317	<0.001	0.002
28	R-mPFC	−0.008 (−0.026, 0.009)	0.009 (−0.009, 0.032)	−3.756	<0.001	<0.001
42	R-DLPFC	−0.005 ± 0.025	0.009 ± 0.026	−3.083	0.003	0.003

CSVD, cerebral small vessel disease; CSVD + S, CSVD with sleep disturbance; CSVD − S, CSVD without sleep disturbance; Oxy-Hb, oxyhemoglobin; R, right; L, left; mPFC, medial prefrontal cortex; DLPFC, dorsolateral prefrontal cortex; FDR, false discovery rate.

### 3.3 Mean Oxy-Hb changes of different brain regions during the VFT

Region-specific differences in mean Oxy-Hb concentration changes were observed between groups (Figure 4C; Table 3). Compared with the CSVD − S group, the mean Oxy-Hb changes in the CSVD + S group were significantly reduced in the R-mPFC (Z = −3.736, FDR corrected *p =* 0.006), L-mPFC (t = −2.315, FDR corrected *p =* 0.044), R-DLPFC (t = −2.439, FDR corrected *p =* 0.048), and L-DLPFC (Z = −2.439, FDR corrected *p =* 0.041). No significant difference was observed in the mean Oxy-Hb changes of the R-TL and L-TL (FDR corrected *p >* 0.05).

**Table 3:** Mean Oxy-Hb changes of different brain regions.

Brain region	CSVD + S	CSVD − S	t/Z	Unadjusted, *p*-value	*p*-value (FDR corrected)
R-TL	−0.002 ± 0.026	0.002 ± 0.030	−0.545	0.587	0.587
L-TL	0.001 ± 0.031	0.005 ± 0.025	−0.724	0.471	0.565
R-mPFC	−0.004 (−0.016, 0.007)	0.011 (−0.007, 0.031)	−3.736	<0.001	0.006
L-mPFC	0.002 ± 0.021	0.013 ± 0.032	−2.315	0.022	0.044
R-DLPFC	−0.003 ± 0.021	0.008 ± 0.027	−2.329	0.016	0.048
L-DLPFC	0.002 (−0.021, 0.010)	0.010 (−0.010, 0.025)	−2.220	0.027	0.041

CSVD, cerebral small vessel disease; CSVD + S, CSVD with sleep disturbance; CSVD − S, CSVD without sleep disturbance; R, right; L, left; TL, temporal lobe; mPFC, medial prefrontal cortex; DLPFC, dorsolateral prefrontal cortex; FDR, false discovery rate.

### 3.4 Relationship between cortical activation with PSQI scores in CSVD patients

**Figure 5:**
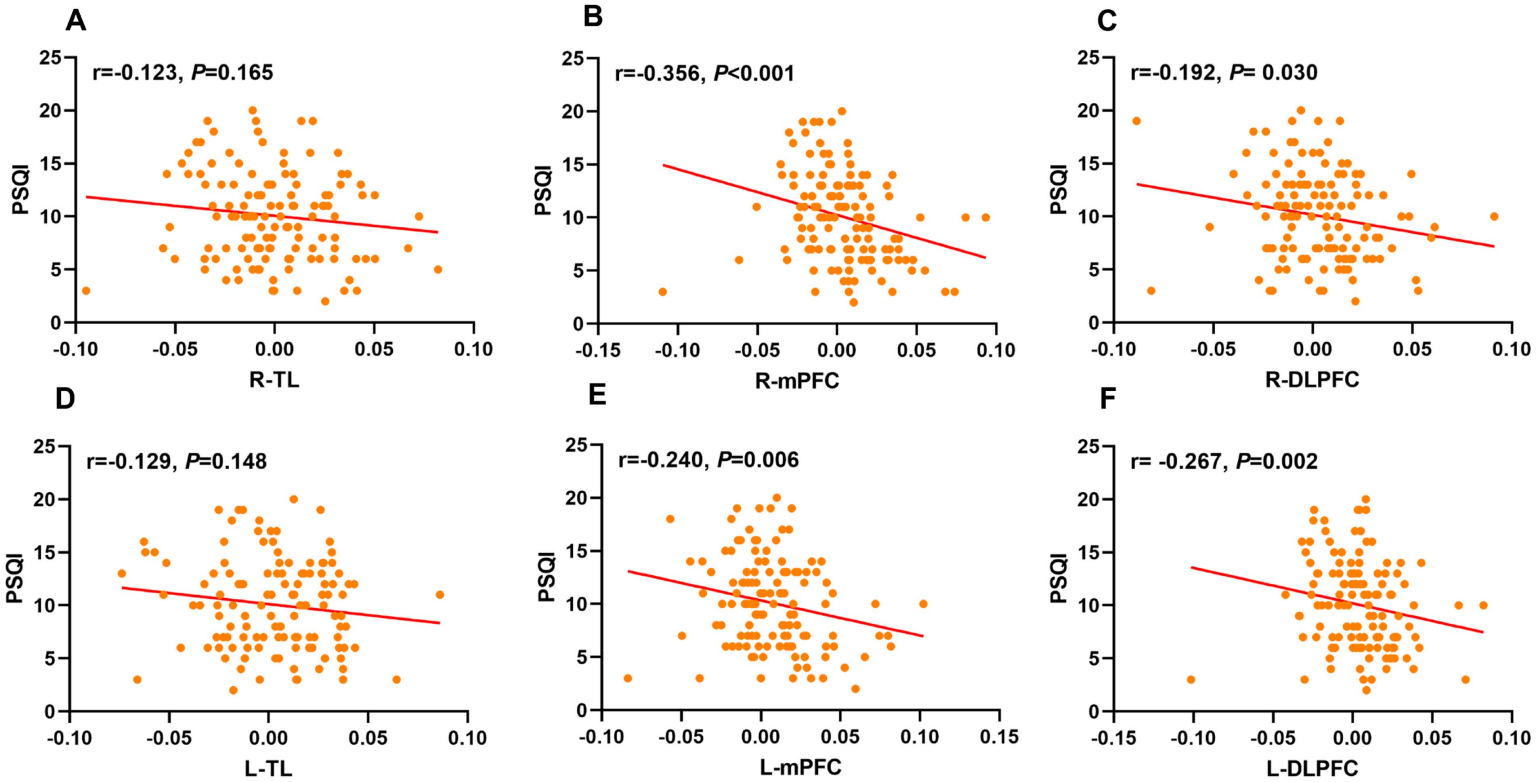
Scatter plots labeled **(A–F)** show correlations between PSQI scores and different ROIs. Correlation coefficients (r values) and p-values indicate varying degrees of negative correlations across different ROIs. Plots **(B, C, E, and F)** show statistically significant correlations. ROIs, regions of interests; PSQI, Pittsburgh Sleep Quality Index; R, right; L, left; TL, temporal lobe; mPFC, medial prefrontal cortex; DLPFC, dorsolateral prefrontal cortex.

Spearman correlation analysis showed significant negative associations between PSQI scores and activation in R-mPFC (r = − 0.356, *p* < 0.001), L-mPFC (r = − 0.240, *p* = 0.006), R-DLPFC (r = − 0.192, *p* = 0.030), and L-DLPFC (r = − 0.267, *p* = 0.002) (Supplementary Table 2; Figure 5). No statistically significant relationship was observed between PSQI scores and activation in R-TL (r = − 0.123, *p* = 0.165) and L-TL (r = − 0.129, *p* = 0.148). Further stepwise multiple linear regression revealed that R-mPFC activation was significant negative correlation with PSQI scores after adjusted for age, gender, education year, CSVD total burden, VFT, and MMSE (*β* = −39.072, t = −2.853, *p* = 0.005) (Table 4).

**Table 4:** Multiple linear regression analysis for the association between cortical activation and PSQI scores in CSVD patients.

Model	Variable	β	Std. Error	t	*p*-value	β (95% confidence interval)
Crude	R-mPFC	−43.063	13.467	−3.198	0.002	−43.063 (−69.459 ~ −16.668)
Model 1	R-mPFC	−39.072	13.695	−2.853	0.005	−39.072 (−65.913 ~ −12.230)
Model 2	R-mPFC	−39.072	13.695	−2.853	0.005	−39.072 (−65.913 ~ −12.230)

Model 1: Adjusted for age, gender, BMI, and education year. Model 2: Adjusted for Model 1 plus CSVD total burden, VFT and MMSE. PSQI, Pittsburgh sleep quality index; R, right; mPFC, medial prefrontal cortex; β: standard regression coefficient.

## 4 Discussion

To our knowledge, this is the first study using fNIRS to investigate the differences in cortex activation of the frontotemporal regions between CSVD + S and CSVD − S groups during the VFT, and further explore its relationship with sleep quality. We found that CSVD + S groups exhibited lower activation in the bilateral mPFC and DLPFC compared to CSVD – S patients. Correlation analysis showed bilateral mPFC and DLPFC activation was negatively correlated with PSQI scores in CSVD patients. Further stepwise multiple linear regression found R-mPFC activation had the strongest association with PSQI scores after adjusting for potential confounding factors.

In our study, the prevalence of sleep disturbance in CSVD patients is 46.01%, which is consistent with previous reports that the prevalence of sleep disturbance in elderly CSVD patients is approximately 46–50% (12, 13), indicating sleep disturbance is common in CSVD patients. In addition, CSVD patients with sleep disturbance have more severe cognitive impairment than those without sleep disturbance, as evidenced by lower MMSE scores and fewer word responses during the VFT. This finding aligns with a previous study indicating that insomnia-induced sleep continuity disruption exacerbates executive dysfunction and memory impairments in CSVD patients (12). Sleep disturbance is closely related to cognitive decline (32) and is considered a partial cause of pathological progression in neurodegenerative diseases such as Alzheimer’s disease (33). This may be due to poor sleep quality can disrupt the clearance of neurotoxins in the brain, impair the flow of interstitial fluid, and affect the space around blood vessels. Therefore, early identification and effective treatment of sleep disturbance might be crucial for improving cognitive function in CSVD patients.

We found that CSVD patients with sleep disturbance exhibited lower levels of activation in the bilateral prefrontal cortical (PFC) than those without sleep disturbance. Two studies using fNIRS-VFT paradigm in patients with chronic insomnia found similar results, reporting a decrease in bilateral PFC activity (21, 34). The VFT is one of the commonly used tasks for evaluating execution function (EF), mainly detecting language production and retrieval. These results indicate the PFC dysfunction associated with EF, which appears to play a role in these patients with sleep disturbance. This may be due to abnormal brain structure in the PFC of insomnia patients. A genome-wide association study (GWAS) found that genes associated with insomnia were enriched in the transcriptional coexpression profile involving the DLPFC and mPFC, suggesting that insomnia may be related to neurodevelopmental abnormalities in brain regions such as the PFC (35). However, a fNIRS study found MDD patients with insomnia showed greater PFC activation during the VFT (36). This may be due to different brain regions depicted and inconsistent study subjects; Therefore, future fNIRS studies are needed to validate our results.

Our results showed that bilateral PFC activation was negatively correlated with PSQI scores in CSVD patients. In particular, activation in the R-mPFC had the strongest association with PSQI scores. This indicates a negative correlation between sleep quality and frontal cortical activation in CSVD patients. Our finding was generally consistent with a previous fMRI study in chronic insomnia patients, which demonstrated that reduced activation of the right anterior PFC was correlated with poorer sleep quality during the emotional Stroop task (37). It is well known that patients with insomnia are specifically characterized by cognitive hyperarousal, manifested as repeated thinking and worry. More and more evidence suggests patients with insomnia have reduced PFC activity related to EF, indicating that the hyperarousal state of insomnia may be due to a decrease in inhibitory processes (37, 38). In our study, if the lower activation of PFC during the VFT indicates cognitive hyperarousal, then the degree of PFC activation may be negatively correlate with the degree of insomnia severity. In addition, it may also be related to brain connectivity networks. The mPFC is a key node of the default mode network (DMN), and reduced mPFC activation may impair functional decoupling of the DMN during sleep (39), leading to poor sleep quality (32).

This study had some limitations. First, the cross-sectional nature of this observational study inherently limits causal inference; Therefore, longitudinal studies are needed in the future to explore the causal relationship between cortical activation and sleep disturbance in CSVD patients. Second, we used the PSQI scale to assess sleep quality. Given the subjective nature of self-reported scales, future studies should incorporate objective measures such as polysomnography (PSG) for validation. Third, although potential confounders were adjusted in the analysis, residual confounding from unmeasured factors such as anxiety and depressive symptoms may have introduced partial bias in the observed associations. Finally, the spatial resolution limitations inherent to fNIRS constrain the detection of functional changes in the fine structure of the brain. In the future, fMRI can be combined with fNIRS for research to more accurately detect frontal lobe dysfunction in CSVD patients with sleep disturbance. Despite the inherent limitations, our findings provide neurological evidence for CSVD patients with sleep disturbance and may provide potential therapeutic targets for the treatment of sleep disturbance in CSVD patients.

## 5 Conclusion

In summary, our fNIRS study found that CSVD patients with sleep disturbance exhibit poorer PFC activation during the VFT, which is linked to poorer sleep quality. This may provide new insights for understanding the pathophysiology of sleep disturbance in CSVD patients and provide potential clinical value for developing treatment strategies for CSVD patients with sleep disturbance.

## Data Availability

The raw data supporting the conclusions of this article will be made available by the authors, without undue reservation.
