# Supplementary material for: Relationship between cortical activation and sleep quality in cerebral small vessel disease patients: a functional near-infrared spectroscopy (fNIRS) study
Source: Front Neurol. 2025 Sep 1;16:1618240. doi: 10.3389/fneur.2025.1618240 (PMC12433872; doi:10.3389/fneur.2025.1618240)
Supplement: Supplementary file 1 [file Data_Sheet_1.docx]

**Supplementary Table 1**. Total cerebral small vessel disease score features and categories

| MRI feature | Visual assessment | Definition | Score | MRI example |
| --- | --- | --- | --- | --- |
| Lacunes | International  consensus  definition (1) | Presence of lacunes ≥ 1 | 1 point | 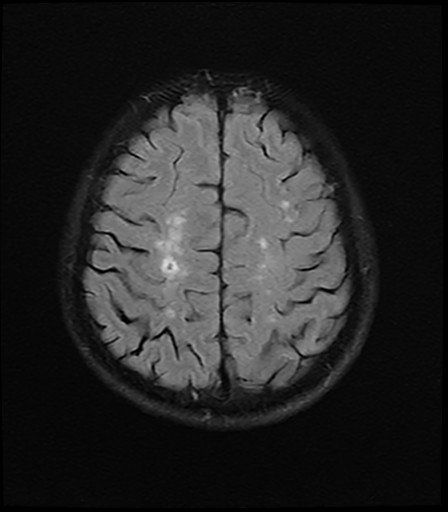 |
| Cerebral microbleeds | International  consensus  definition (1) | Cerebral microbleeds ≥ 1 | 1 point | 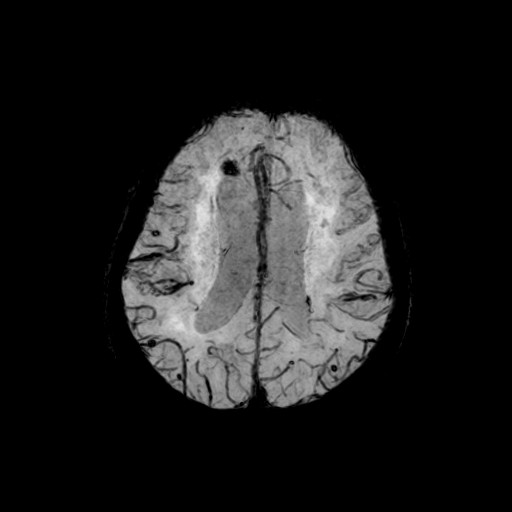 |
| Enlarged perivascular spaces | Semiquantitative scale (2) | Moderate-to-severe enlarged perivascular spaces in the basal ganglia | 1 point | 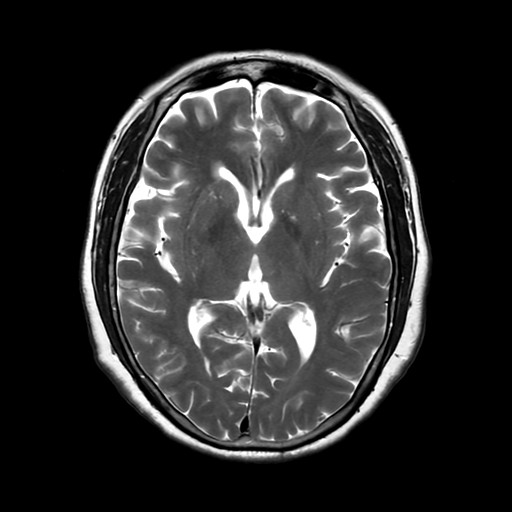 |
| White matter hyperintensities (WMH) | Fazekas scale (3) | deep WMH graded as Fazekas score 2 (beginning confluence) or 3 (confluent lesions), or irregular periventricular WMHs with deep white matter extension (Fazekas score 3) | 1 point | 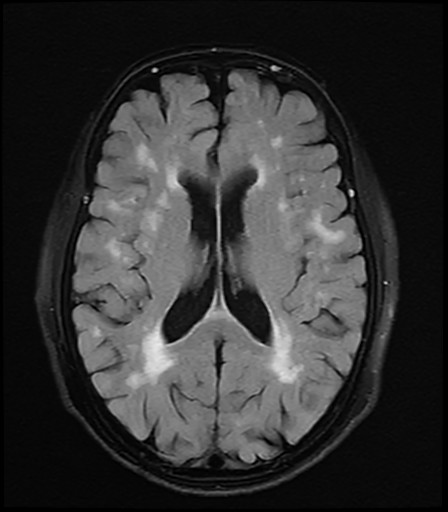 |

# Reference

1. Wardlaw JM, Smith EE, Biessels GJ, Cordonnier C, Fazekas F, Frayne R, et al. Neuroimaging standards for research into small vessel disease and its contribution to ageing and neurodegeneration. *Lancet Neurol*. (2013) 12:822-38. doi:10.1016/s1474-4422(13)70124-8.

2. Doubal FN, MacLullich AM, Ferguson KJ, Dennis MS, Wardlaw JM. Enlarged perivascular spaces on MRI are a feature of cerebral small vessel disease. *Stroke*. (2010) 41:450-4. doi:10.1161/strokeaha.109.564914.

3. Fazekas F, Chawluk JB, Alavi A, Hurtig HI, Zimmerman RA. MR signal abnormalities at 1.5 T in Alzheimer's dementia and normal aging. *AJR Am J Roentgenol*. (1987) 149:351-6. doi:10.2214/ajr.149.2.351.

**Supplementary Table 2**. Correlations of cortical activation with PSQI scores in CSVD patients.

|  | PSQI | |
| --- | --- | --- |
|  | r | *p*-value |
| R-TL | -0.123 | 0.165 |
| L-TL | -0.129 | 0.148 |
| R-mPFC | -0.356 | **<0.001** |
| L-mPFC | -0.240 | **0.006** |
| R-DLPFC | -0.192 | **0.030** |
| L-DLPFC | -0.267 | **0.002** |

Abbreviations: CSVD, cerebral small vessel disease; R, right; L, left; TL, temporal lobe; mPFC, medial prefrontal cortex; DLPFC, dorsolateral prefrontal cortex; r, spearman correlation coefficient.
